# The phenomenological model of depression: from methodological challenges to clinical advancements

**DOI:** 10.3389/fpsyg.2023.1215388

**Published:** 2023-10-31

**Authors:** Oskar Otto Frohn, Kristian Moltke Martiny

**Affiliations:** The Enactlab, Copenhagen, Denmark

**Keywords:** depression, phenomenological psychopathology, phenomenological interview, patho-description, affordance

## Abstract

In this article our overall aim is to illustrate how phenomenological psychopathology can advance the clinical work on depression. To do so, we start by unfolding the current phenomenological model of depression. We argue that this model faces a methodological challenge, which we define as ‘the challenge of patho-description’. Mental disorders, such as depression, influence how people are able to access and describe their own experiences. This becomes a challenge for phenomenological psychopathology since its methodology is based on people’s ability to describe their own experiences. To deal with this challenge, in the case of depression, we turn to the framework of phenomenological interview. We interview 12 participants (7 women, 5 men, age-range from 29 to 57 years) with moderate and severe depression. From the interview results, we show how phenomenological interview deals with the challenge of patho-description and how patho-description in depression conceals experiential nuances. We unfold these nuances and describe how people with depression pre-reflectively experience a variety of feelings, a type of agency, overly positive self-image, and relations in a hyper-social way. These descriptive nuances not only strengthen the phenomenological model of depression, but they also help advance the clinical work on depression. We firstly illustrate how the descriptive nuances can be added to current manuals and rating scales to advance diagnostic work. Secondly, we illustrate how phenomenological, ‘bottom-up’, and embodied approaches function at the pre-reflective level of experience, and that further effort at this level can help advance therapy for depression.

## 1. Introduction

The research landscape of psychopathology has in the last decades been criticized for lack of advancement and not delivering on the promise to provide better quality care for people with mental disorders (Thornicroft, 2007; Stephan et al., 2016; Leichsenring et al., 2022; Stein et al., 2022). This calls for a reformation in the field of psychopathology (Bizzari, 2019). One contribution to such a reformation is the tradition and discipline of Phenomenology.

Over the past century, prominent psychiatrists such as Karl Jaspers (1883–1969), Ludwig Binswanger (1881–1966), and Eugene Minkowski (1885–1972) have sought to incorporate phenomenology with work in psychopathology.^1^ Fundamental to phenomenological psychopathology is the critique of the dominating biomedical model of psychopathology and its conformity to the method of operationalism (Schwartz and Wiggins, 1985; Mullen, 2007; Nordgaard et al., 2013; Parnas et al., 2013; Sass, 2022). The longing for objectivity and reliability seen within operationalism has led to the notion that psychopathological symptoms are explainable in biological terms, and that the field of psychopathology is therefore reducible to the field of biomedicine. However, the symptoms within the two fields differentiate in nature, making symptoms in psychopathology irreducible to somatic dysfunctions (Marková and Berrios, 2016). This means that the epistemological criteria within the two fields are not transferable (Zahavi and Parnas, 2009).

This criticism of the reductionistic and objectivistic tendencies seen in operationalism indicates that a reformation in the field of psychopathology should include more nuanced understanding of psychopathological symptoms by including the subjective experience of mental disorders (Ratcliffe and Broome, 2012; Ratcliffe, 2015; Stephan et al., 2016; Brodersen, 2017; Sass et al., 2018; Aragona, 2020; De Haan, 2020b; Ritunnano et al., 2023). In contrast to operationalism, phenomenological psychopathology takes its point of departure in people’s lived experience with the aim of integrating body, mind, and world into a holistic model (Fuchs, 2013; Ratcliffe, 2015; Marková and Berrios, 2016; Zahavi and Martiny, 2019; De Haan, 2020a,b). The overall aim is to bridge the gap between the subjective and objective approaches to psychopathology and understand mental disorders through lived experience – avoiding the risk of reductionism and oversimplification (Parnas and Gallagher, 2015). This means we need an approach that does not reduce, but in fact emphasizes the nuances and complexities of subjectivity, the first-person point of view, and conscious experiences (Parnas et al., 2013).

The broad consensus, that the dominating approach in psychopathology lacks advancements, has helped the field of phenomenological psychopathology to flourish throughout the last two decades. Some of the most impactful insights in phenomenological psychopathology come from the clinical work on schizophrenia and is exemplified through the ‘Examination of Anomalous Self Experience’ (“EASE”) protocol (Parnas et al., 2005). EASE has shown how the field of psychopathology and psychiatry can benefit from phenomenology. By combining a semi-structured qualitative interview design with a semiquantitative psychometric checklist, EASE provides a way to work with the nuances and complexities of the patients’ subjective experience as a supplement to standardized diagnostic systems (e.g., ICD and DSM). EASE therefore produces rich and nuanced descriptions that are invaluable in diagnosing and understanding schizophrenia.

The current problem is, however, that unlike EASE, most work within phenomenological psychopathology has gained little traction and impact within clinical and teaching settings (Andreasen, 2007; Ritunnano et al., 2023). The resources and insights coming from phenomenological psychopathology have not been widely integrated, translated, nor applied in clinical research and practice. To fully show the value that phenomenology can provide to psychopathology and psychiatry, we need to show how many different cases, resources, and insights can lead to clinical advancements. As Messas and Fernandez (2022) argue: to fully develop the approach of phenomenological psychopathology, we need to develop additional paradigm cases, other than schizophrenia, for scholars and healthcare practitioners to be inspired by.

In this article we will look at the case of depression to further explore the strength of phenomenological psychopathology. We will start by unfolding the current phenomenological model of depression and emphasize a methodological challenge that the model faces. To deal with this challenge, we turn to the framework of phenomenological interview. Based on phenomenological interviews with 12 persons with moderate and severe depression, we show how the phenomenological interview deals with the methodological challenge and how the results can help nuance the phenomenological model of depression to further advance the diagnostic and clinical work with depression.

## 2. The phenomenological model of depression

There is already extensive and complex phenomenological literature on the experience of depression, so in the following we will not be able to give a full account of this literature. However, we will structure the work into what can be defined as a ‘phenomenological model of depression’. We do so by combining phenomenological psychopathology with the ‘bio-psycho-social’ model (Engel, 1977), as it has been done in a few other cases (Sass et al., 2018; De Haan, 2020b). To make clear delimitations we therefore divide the experience of depression into four phenomenological dimensions: the existential, biological, social, and psychological.

### 2.1. The existential dimension

The existential dimension in depression refers to the way in which people relate to and make sense of themselves and their situation. It concerns the underlying structure that enables one to fundamentally feel something and belong to a meaningful world. When living with depression the existential dimension is affected through the loss of immediate meaning. One’s sense-making and stance-taking has been disturbed, and the way in which one perceives and understands one’s world no longer makes sense (De Haan, 2017, 2020b).

To borrow a term from Ratcliffe (2015, 2020), in depression the *existential feelings* that are pre-intentionally given and provide a feeling of belonging in the world are disrupted. These feelings structure our sense of reality and situatedness in the world and when disrupted people feel as though they ‘live in a different world’. The overall structure of world-experience is altered, where everything looks the same yet is experienced completely different (Ratcliffe, 2005, 2015; Ratcliffe and Broome, 2012). The experience of the world loses its ‘sense’ and ‘feeling’, and the existing experiences can be described as ‘deprived of meaning’, ‘estranged’, ‘detached’, and ‘alienating’ (Fuchs, 2013; Ratcliffe, 2015).

Overall, the experience of depression can therefore be described as a ‘feeling of loss of feelings’ (Jaspers, 1963; Stanghellini, 2004), or as it has been paradoxically described by patients: a ‘feeling of not feeling’ (Fuchs, 2005).

### 2.2. The biological (embodied) dimension

Firstly, when considering the biological dimension, it’s important to emphasize that the term ‘biological’ is misleading and may refer to the biomedical understanding of depression.^2^ In the context of the phenomenological model of depression the term ‘biological’ should rather be understood in terms of a person’s embodied, somatic, and corporeal relation to the world.

Depression alters the person’s embodied spatial and temporal experiences, which can be understood through disembodiment (Fuchs, 2013). The world is no longer experienced as a horizon of possibilities (Minkowski, 1970; Ratcliffe, 2015), there is no motivation for actions, nor initiation, and overwhelming sensorimotor inhibition takes place. This disembodiment is also described as a *corporealization* of the lived body. Here the constriction of bodily potentiality and capability results in the hindrance of temporal movement as the future is experienced as static, future actions seem inaccessible and hopelessness becomes pervasive. This ultimately leads to a desynchronization of common time, otherwise socially constituted (Fuchs, 2001, 2014; Wyllie, 2005). As a consequence, a person with depression must push herself to act, even with the smallest tasks (Fuchs, 2005, 2007, 2013). Depression can therefore be described as an impaired ability to act (Ratcliffe et al., 2013), a diminished sense of agency (Ratcliffe, 2015), or an impaired agency (Slaby et al., 2013).

One way to further clarify this disembodiment in depression is through the ‘theory of affordances’ (Gibson, 1979). Usually, we live through our body, focused on the world, where objects and situations are experienced both as ‘ready-to-hand’, affording a range of action possibilities, and as having emotional value and relevance (e.g., important, joyful, fearful, or disgusting). The term ‘affective affordances’ (Fuchs and Koch, 2014) clarifies that we experience the world through an emotional ‘pull’, which is part of our motivation for action. However, in depression there is lack or loss of affective affordances. The body is experienced in a hyper-objectified or quasi-mechanical way, where the body is felt as if it has lost its affectivity, fluidity, mobility, and flexibility (Krueger and Colombetti, 2018). The field of affordances no longer solicits actions, and the person becomes incapable of engaging with the world in a spontaneous and lived manner (Aho, 2013). As Krueger and Colombetti (2018) point out, the distortion of the field of affordances in depression is extensively filled with anomalous experience of embodiment and body-schematic disturbances (see also Doerr-Zegers et al., 2017).

**Figure 1:**
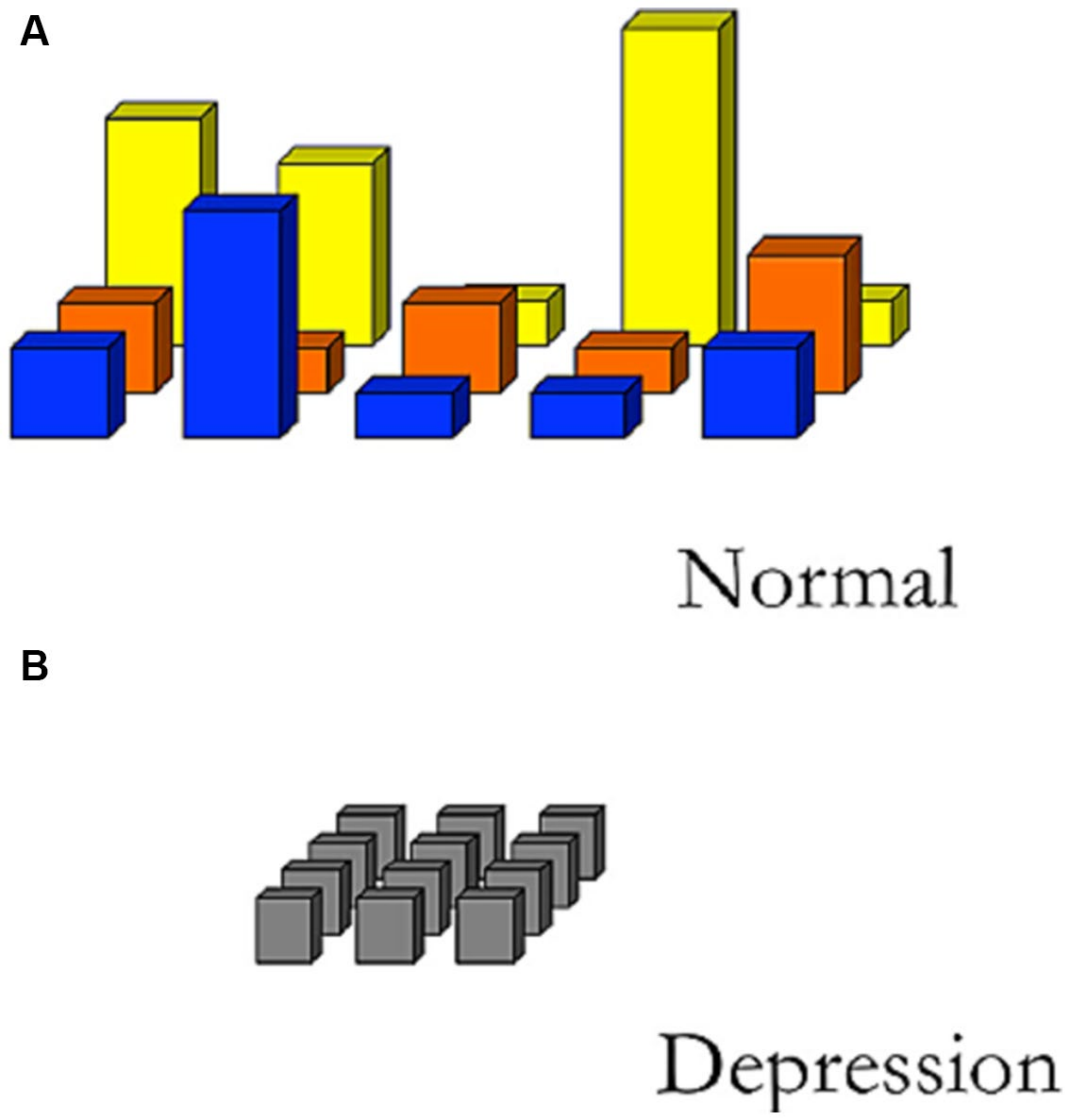
Sketch of different fields of relevant affordances. **(A)** Illustrates how emotions and moods of a normal person provide a differentiated field of affordances, whereas **(B)** due to the affective disturbances in depression the field of affordances is rather flat where nothing stand out to solicit actions (De Haan et al., 2013).

The typical depiction of the field of affordances in depression is therefore as an emotionally flat, grey field, where nothing stands out. There is so to say a reduction in the ‘phenomenal depth’ (Church, 2003; Gaebler et al., 2013) of the experiential life in depression, where experiences have lost their feeling, affective affordance, and action possibilities. Figure 1 illustrates such depiction where the field of affordances for ‘normal’ people (1A) is contrasted with the field of people living with depression (1B) (De Haan et al., 2013; Bruineberg and Rietveld, 2014).^3^ In Figure 1B the ‘grayness’ of the field emphasizes the experience of loss of feelings and meaning in depression, the ‘height’ emphasizes the reduction of the relevance and salience of affordances, and the ‘width’ and ‘depth’ emphasize the reduction of action possibilities (both in space and time).

### 2.3. The social dimension

Taking a point of departure in the understanding of embodiment in depression, Fuchs (2013) describes the social dimensions of depression by referring to a ‘detunement’ of people’s interaffectivity and interpersonal encounters. Usually, we are both positively and negatively affected by our surroundings, environment, and by the bodies and emotions of other people. However, due to the loss of embodied connectivity to the world, a person with depression is interaffectively detuned. This means that while positive affections are diminished in social interactions, negative affections are heightened. Negative feelings such as guilt and shame permeate the social life of the person with depression, to an extent that they become existential background feelings (Ratcliffe, 2005, 2015; Škodlar and Henriksen, 2019). This also means that Figure 1B seems to be omitting certain aspects of depression, since a variety of negative feelings are dominating the social life in depression, which is not depicted in the figure as part of the field of affordances. We will further discuss and develop Figure 1 below (see § 5.3).

In the phenomenological model of depression, the social dimension connects to the psychological dimension, since the despair over themselves and their social inabilities becomes a negative spiral that keeps people in their depression. The result is that the person feels like an “isolated object in a world without relationships” (Fuchs, 2013, p. 229), and she isolates herself from the world. Unable to find one’s place in the world, Tellenbach states that “it is the relation of the “I” to itself that is threatened in depression” (1980, p. 166). In other words, in the context of other people a person with depression will find her perception of herself threatened.

### 2.4. The psychological dimension

When discussing the ‘I’ within depression there are two personality types prone to depression that can be highlighted as the most frequent variants of personalities (Zerssen and Pössl, 1990; Ronningstam, 1996; Mundt et al., 1997; Kronmüller et al., 2002; Stanghellini et al., 2006; Twenge and Campbell, 2008; Twenge and Foster, 2010; Fuchs, 2019). These types are the *Typus Melancholicus* (Tellenbach, 1980) and the narcissistic personality type (Ehrenberg, 2010).

The *Typus Melancholicus* is defined through rigidity, conscientiousness, orderliness, over-adaptation, and over-identification with social norms and roles, as well as being overly dependent and even symbiotic in relations (Tellenbach, 1980). Creating a rigid world in which they operate, the identity of people with this personality type is fixated upon a dictated social role. If this non-autonomous role is destabilized through falling “short of their duties, if they experience unjustified rejections or the loss of significant others, then their world literally collapses.” (Fuchs, 2019, p. 619). In such cases, feelings of guilt for not living up to one’s expectations become existential. This means that the experience of guilt becomes pervasive, and the individual will feel as though they are living in a different world, shifting the overall structure of world-experience, where everything looks the same, yet is experienced completely different (Ratcliffe, 2005, 2015; Ratcliffe and Broome, 2012).

In contrast, the narcissistic personality type is defined by a person’s constant need for affirmation by her environment and others. This is done to maintain a prevailing self-image so idealized that it captivates the individual, potentially leading to impotence and paralyzation (Ehrenberg, 2010). It’s an existence full of shame, since in her fundamental megalomania a person with depression cannot accept being limited by reality, thereby severely disabling herself by setting unachievable goals that eclipse her very own finite capabilities (Micali, 2019).

Pertaining to both types of personalities is a disruption of the person’s narrative identity (Bortolan, 2017). Usually, when we create and tell stories about ourselves, there is an authenticity to the narratives that are based partly on the values that we identify with and the affective experience that counterparts these narratives. In depression the person becomes estranged from the values that she used to identify with, and the narratives lose their affective feeling (see also Stanghellini, 2004). Instead, as Bortolan (2017) argues, the person with depression creates new narratives that are based on existential feelings such as guilt, hopelessness, and isolation.

This means first of all that people with depression create their new narratives by adopting and adapting stories created by others into their own narratives. Their creation of new narratives is therefore dictated by the scripts of their social and cultural environment, and by how other people tell stories about depression. Secondly, it means that the narratives can be characterized by ‘over-general descriptions’ of the ‘same old stories’ about the interpersonal and emotional life of people with depression (see also Angus and Greenberg, 2011). There is therefore a repetitive nature to their storytelling. Thirdly, it means that there is an ‘explanatory style’ that people with depression use when they interpret and explain events that have happened in their life. In depression this style is ‘pessimistic’, where people with depression tend to conceive of events in negative terms. They will personally take responsibility for the event; see it as something permanent, pervasive, and universal, rather than temporary. In other words, their style features negative and permanent causal explanations, and a person with depression will explain their failures through character traits.

### 2.5. A methodological challenge

As with much work within phenomenological psychopathology, the phenomenological model of depression has not been widely integrated, translated, nor applied in clinical research and practice. There are different reasons for this, of course, but one of the reasons is at the heart of phenomenological psychopathology. It concerns how we work with subjectivity and study the lived experience of, for example, people with depression, without undermining the necessary rigor needed for research in psychopathology.

The longing for objectivity seen with operationalism is partly fueled by the worry of how we deal with subjectivity within research. Such worry has been discussed extensively in conversations about for example ‘cognitive bias’ (Tversky and Kahneman, 1974; Nisbett and Wilson, 1977). It is challenging to include people’s reports and descriptions of their own experiences, because how do we know if their description reflects their lived experiences?

As we have seen with the case of depression, the way that people create narratives and describe their experiences is part of the psychopathology. There are also other cases within psychopathology (e.g., schizophrenia), which cause the absence of self-awareness of the disorder, making people unable to describe aspects of their experiences (Parnas et al., 2021). And there are cases in healthcare such as in living with disability (e.g., cerebral palsy), where people adopt the technical language and explanations from healthcare as part of their own descriptions and self-narratives (e.g., “according to my physician I experience disability because I have a brain damage in my motor cortex”; Martiny, 2015b; Høffding and Martiny, 2016). More generally, people living with health challenges, illnesses and disorders also create self-narratives that can be used as coping strategies (Kelley and Clifford, 1997; Tighe et al., 2011; Carel, 2021). Thus, in using descriptions to study subjectivity and lived experience within healthcare and psychopathology, we must understand how the disorders, illnesses, disabilities, and health challenges influence the way that people describe their experiences.

Influences and cognitive biases are a methodological challenge for all research studying subjectivity and experience using descriptions and self-reports, but in the context of psychopathology and healthcare we will define it as ‘the challenge of patho-description’:

*Living with a disorder, an illness, a disability, or a challenge to one’s health influence how people are able to access and describe their own experiences.*

The challenge here is not a question of whether people are able to give accurate descriptions of their experience. It’s a methodological question of how we as researchers deal with the fact that the way people are able to access and describe their own experiences is influenced by their disorders, illnesses, disabilities, and health challenges. For example, in working with people’s descriptions of their experience in psychopathology, how do we differentiate between descriptions that reflect their symptoms, and descriptions that reflect disturbances in narratives, coping strategies, or absence of self-awareness?

The challenge of patho-description questions the methodological foundation upon which the current phenomenological model of depression has been developed. As seen in most of the research literature, the model has been developed on literary and autobiographical descriptions (Rowe, 1978; Styron, 1992; Wolpert, 2006; Brampton, 2008), subjective reports to questionnaires, interview descriptions, and clinical conversations with people with depression. And there certainly are a lot of crucial insights, learnings, and resources within this research. However, as we have argued, the challenge of patho-description is fundamental to how people with depression create narratives and describe their experiences. This means that simply relying on the words, narratives, and descriptions coming from people with depression is methodologically problematic. The descriptions that they will provide of their experiences might be over-generalized and pessimistic, telling the same negative stories, which they see as permanent, pervasive, and universal. As a result, the descriptions might conceal experiential nuances fundamental to the understanding of depression.

In the rest of this article, it is our aim to deal with the challenge of patho-description in the case of depression, and disclose some of these experiential nuances. We do so by turning to the framework of the phenomenological interview.

## 3. Materials and methods

One approach that has been highly successful in working with subjectivity and lived experience within healthcare and psychopathology is the methodological frame of the phenomenological interview (Høffding and Martiny, 2016; Martiny et al., 2021). Phenomenological interview is one of the core elements in EASE (Parnas et al., 2005), and it has been successfully applied in different psychiatric settings (Nordgaard et al., 2013), and in other settings such as in work with disability (Martiny, 2015a,b). Overall, the phenomenological interview is a systematic frame that deals with the methodological challenges of working with subjectivity and experience, which can then be applied within a healthcare and psychopathological context (Zahavi and Martiny, 2019).

### 3.1. The phenomenological interview

The phenomenological interview is a specific type of qualitative interview that is based on the scientific foundation of philosophical phenomenology with its specific methodological commitments and conditions. This means that it operates with specific second-person interview questions and techniques, as well as with a specific phenomenological analysis strategy (Høffding and Martiny, 2016; Martiny, 2017; Zahavi and Martiny, 2019; Martiny et al., 2021). It is this framework of commitments, conditions, techniques, and strategies that makes the interview “more like science than like psychotherapy” (Gallagher and Zahavi, 2008, p. 28), and provides a solution to the challenge of patho-description.

As such, the aim of the phenomenological interviews is not just to understand what a particular person with depression is experiencing here and how. Descriptions of such idiosyncratic experience would succumb to the challenge of patho-description, as described above. The aim of the phenomenological interview is rather to capture the invariant structures of the experiences. This means that the phenomenological framing of the interview ensures that the data generation and analysis of the lived experiences go beyond the idiosyncratic, and for example the coping strategies, or the specific explanatory and descriptive style seen in depression. It does so, by working with specific interview questions and techniques, which have been thoroughly described in Petitmengin (2006), and by working with structural analysis of the interview descriptions, which have been thoroughly described in Høffding and Martiny (2016) and Martiny et al. (2021).

In this study of the lived experience of depression, we used the phenomenological interview as the frame to develop an interview guide and analysis strategy. Taking a point of departure in the current phenomenological model of depression, we front-loaded phenomenological concepts (e.g., (inter)affectivity, (inter)corporeality, and agency) and distinctions (reflective/pre-reflective experience and minimal/narrative self) into the interview design, structure, and questions. We also structured the interviews in four overall themes focusing on the four phenomenological dimensions of depression: (1) the existential dimension, their emotions and feelings, (2) the biological (embodied) dimension, their agency and bodily activities, (3) the psychological dimension, their understanding and narratives about themselves and their identity, (4) the social dimension, their social life and being together with other people.

To elicit detailed descriptions the questions were structured as open ‘how-questions’, focusing on concrete experiences and situations within these four themes. This was done to avoid overgeneralized explanations, biases, assumptions, and common stories about depression. Focusing on the episodes when they experience depression, the questions included for example: “How do you experience a typical morning?,” “Can you describe the feelings and emotions that you experience when you have to get out of bed in the morning or when you have to go to bed at night?,” “How do you experience doing daily activities such as cleaning up, doing the dishes, or buying food?,” “How do you experience being with other people: At home with family members, at a party with friends, with colleagues at work, or classmates at school?”

To analyze the descriptions from the interviews within a phenomenological interview frame we first transcribed the recorded interviews. From the transcriptions we coded the data and structured the data to identify experiential categories that correlate with the front-loaded phenomenological concepts and distinctions. The analysis was done in Danish and then translated to English. Few challenges arose while translating the descriptions from Danish to English. One of them concerned the participants frequent usage of the Danish word ‘man’. ‘Man’ has an implied normative aspect to it, and to capture this, we have chosen to translate it to ‘one’ to explicitly express the normative aspect.

### 3.2. The participants

We recruited 12 Danish participants (7 women and 5 men) in the age-range from 29 to 57 years that all had been diagnosed with moderate or severe depression and had experienced one episode or several episodes with depression. The recruitment criteria clarified that the participants should be clinically diagnosed with depression (moderate or severe), be older than 18 years, not be currently going through a depressive episode, and be emotionally stable enough to answer questions as those described above.

The recruitment was done in collaboration with the Danish Depression Union and the NGO ‘En af Os’ (eng. ‘One of Us’) that work with and represent people with depression among other mental disorders. In the start of the recruitment process, we were only able to recruit women. After we got 7 women to participate, we shifted our strategy and only recruited men to get enough recruitment variation. In order to participate the participants had to read and sign a consent form. The interviews were either conducted at the University of Copenhagen or at the participant’s own home, depending on their preference. The interviews took between 1 and 2 hours, were recorded, and all the names of the participants’ have been anonymized for the sake of privacy.

## 4. Results

The results are categorized within the four phenomenological dimensions and themes, and an overview of the results is presented in Table 1. The table presents the descriptive data, where a selection of quotes exemplifies the codes and categories within which the experiences have been analyzed. In the following the results will be briefly described.

**Table 1:** Experiences of depression.

Category	Codes	Descriptions
1. Theme: Existential Dimension (Feelings and Meaning)		
Loss of feeling and meaning	Lack of meaning	1A: “I experienced that I became more and more apathetic toward my own life... Even the things that I thought were fun normally, and which gave me something normally - it was all devoid of meaning” (Man, 32)
	Lack of energy	1B: “There is no interest, whatsoever, for what I used to be interested in. It is kind of like wearing an old-fashioned diving suit, that you walk around in. Everything is heavy, there is lead in your feet, and I use my remaining energy on taking these steps. So, there is a deep feeling of discouragement, loneliness, and fatigue - absolutely drained of energy.” (Woman, 33)
	Distortion of reality	1C: “When you lose contact with the feel of right and wrong, good and bad, then you develop these antennas for what other people want… Then I do not see what is real… All I see is a negative reflection of myself.” (Woman, 39).
Emotional variation	Day-variation	1D:“The emptiness is the most dominating feeling, but after that, there is this day-variation, then the feelings start coming throughout the day. So gradually, as I become more and more active, then I can think different thoughts and feelings.” (Woman, 57)
	Anger	1E: “…my condition can change from one moment to another, or it could then. And if someone said something wrong to me, then I got mad. I should preferably be agreed with, otherwise I would get angry and aggressive. And the rage accumulated within me…” (Man, 49)
	Sensitivity	1F: “It feels physical and psychological at once. I have no resistance to cold, heat, thoughts, inner feelings – it’s like… Imagine you have no skin, and someone touched you, and it would hurt, sting, and burn – everything just hurts, and everything was uncomfortable, because I just had no skin on my body…” (Woman, 32)
	Humor	1G: “Still today there are many who do not understand that you can laugh even if you have depression… I can because I am very focused on dark humor and irony. I can be laughing at myself, at the situations that I end up in.” (Woman, 44)
2. Theme: The Biological (Embodied) Dimension (Embodiment and Agency)		
Lack of agency	Petrification	2A: “It is not that I am sad, but I am just empty. And infinitely sad, and I feel like that in all my veins there is concrete. And I cannot even move myself, even if it hurts. Often you need to pee in the morning, but I cannot do that, because I cannot go out and pee… it is a petrification.” (Woman, 57)
	Struggle	2B: “I want life, that’s what the struggle is about, but on the other hand, I just do not have the strength for it.” (Woman, 33)
	Tasks	2C: “I cannot even be bothered to brush my teeth, everything is a huge task – and when you have children, then you have to get going.” “Even something so banal as getting a glass of water is such a big task, that you would rather sit and stare into nothing.” (Woman, 44).
	Inability to act	2D: “It’s like being trapped in one’s body. There is some energy, that just cannot amount into anything. It cannot be channeled into action. It is very much ‘should’ and ‘have to’. They say that I will be feeling better if I do this, then I should also be doing something myself, then I also should be making dinner. But then comes this restlessness that I could do something too.” (Woman, 32)
A sense of agency	Liberation	2E: “[I] get going in the morning, get the boy to day-care, and then go all the way to work, without there being any issues [*I: How were you able to do all of these things?*] It was a liberation for me because in the train to work I had a space of freedom. One hour each way.” (Man, 49)
	Suicide	2F“I thought about [suicide] a lot. I thought about how and it was like a valve, where I felt like I was in a situation, where I could not do shit, I could not control anything […] and it was horrible, and here was something that I could do! I could choose not to be here anymore, and it helped… So for me [the suicidal thoughts] have not been so bad. For me, it has just been an action for possibility in something where I felt I could not do anything at all.” (Woman, 39)
		2H“[I: Have you written suicide letters?] Yes, those I have a lot of! I went on a forum, where I discovered, that you could get these things down on paper. And how productive I was, gosh! [I: You had difficulties getting a glass of water, but you were able to write suicide letters and poems?] Yes, that I was able to do. About how terribly I had it, or about how terribly I used to have it.” (Woman, 44)
3. theme: The Psychological Dimension (Self-experience and Narrative)		
Ideal self	Self-image	3A: “When I had it the worst, I saw myself as an Übermensch. It sounds incredibly narcissistic, and it is of course. But in the sense, that I should be better than others – and not in a bookish nor athletic manner. But more in a moral way. I should act good, because I should be a good person. And the conceptions I had, about what I should do and how to behave to be a good person, they were completely unrealistic.” (Man, 24)
	Self-expectations	3B: “It is because, I think, that one has to. There are some expectations, that I must meet. And these expectations aren’t even that bad.” (Man, 24)
	Perfectionism	3C: “If anybody saw that all of it wasn’t perfect, then the world would end.” (Woman, 39)
Shaming self	Self-value	3D: “I connected my value as a person to my work. And when I had to realize, that I actually could not do it, then it all snapped completely, because then I had no motivation to fight.” (Woman, 39)
	Self-conflict	3E: “I had a greater self-image than my self-evaluation could meet or recognize in the social interactions. Probably also why I preferred to not have these interactions, because then I did not have to make those choices, and then I would not have to do the right thing. Then it was better to not be confronted with the fact, that I could not figure out how to be a good person.” (Man, 24)
	Performativity	3F: “Performance. It’s a performance to exist…It is the core of the disease that I should pull myself together. This is connected to the fact that I am what I perform. So if I do not perform anything, then I am nothing. And then I should pull myself together, because if I want a value, then I have to do something too. And why should I not be able to do anything when all other people do it every day and get up and go to work - what is it with me that makes me unable to pull myself together? (Woman, 33).
	Guilt	3G: “In my head, people have a lot of thoughts about how bad of a person I am. And everything that I do is wrong. It is a feeling of never being able to be myself, and quickly overthink and overanalyze even tiny things.” (Woman, 33)
4. Theme: The Social Dimension (Social relations)		
Social conflicts	Social inadequacy	4A: “Then I am even more focused on what others might think. As soon as there is another person, it does not matter what they see, it just is not good enough. It is an inadequacy, and I have them with me under the duvet.” (Woman, 44)
	People-anxiety	4B: “[…] The head is not petrified, because it is alert about everything that happens around oneself. It is the volume of everything, it is just too much… [*I: What is it that takes up so much thought?*] People. Because I know they are right outside the door.” (Woman, 57)
	Social obligations	4C: “…[an employer] had been so good to take me in, when it was really hard. And then you feel obligated. I have a strong moral character… If there is someone helping me, then I feel compelled to help them… that’s how I feel, that there are some specific ways one ought to be… So I feel society does a lot for me, so one has to give everything back, that one can.” (Man, 33)
	Social burden	4D: “In that moment, where I completely gave up on it all. I thought that I was a burden to my family... So, I sent an e-mail about how it wasn’t their fault. Because they should not go around with a feeling of guilt for not helping, because it was me, it was my problem. It was important for me to tell them that.” (Man, 49)
Social connection	Social consideration	4E: “I have fantasized a lot about my own funeral, which there is a lot of love connected to. Later it came to planning [of the suicide], and how considerate it could be done; how one could do it, and where [the suicide] was rather tidy, so people aren’t traumatized. […] Who would receive suicide letters, what should be written in them. I had to consider organ donation, so it would not be too much of a burden.” (Woman, 32)
	Social comparison	4F: “If [my friend] can, then I should be able to as well. What is it that I lack, that others have? I thought that something was wrong with me, that it was my own fault.” (Woman, 32)
	Meaning in others	4G: “There is nothing that is meaningless, when you are picking up a toddler in nursery. They run toward you, and then you have to go to the supermarket and get groceries.” (Woman, 57)
	Dilemma of social relations	4H: “Social situations are so tough. Because by putting yourself out there in the social situation, can be the cause for you breaking, but it can also be the cause to look better at the situation. Which is the hardest – to give others a chance.” (Woman, 44)

Table shows a schematic account of the descriptions given by the participants, coded, and categorized under the heading of the four experiential themes.

### 4.1. The existential dimension: feeling and meaning

When asked about their experiences with depression the participants describe the experience as ‘petrification’, ‘having concrete in the veins’, ‘a black hole’, and a ‘zombie-mode’. Many of their descriptions center on the inability to feel and the loss of meaning (quote 1A).

When asked to elaborate on their experiences, all the participants describe the experience using terms such as ‘grim’, ‘dark’, ‘empty’, ‘sad’, ‘hopeless’, ‘thoughtless’, and ‘nothingness’: “…the experience there in the deepest depression, there is nothing. It’s empty. And there is darkness.” (Woman, 39). The participants describe a distortion of a sense of reality, giving them the feeling of being separated from the world. A separation that can be related to the feeling of apathy (quote 1B), but which also colors the world with negativity and makes it difficult for them to understand and relate to others (quote 1C).

Especially the mornings were experienced as “the worst about a depression, the pain that one has in the morning.” (Woman, 57). However, the participants describe how feelings and meaning gradually become more apparent throughout the day (quote 1D). In addition, participants describe constant changes in moods and feelings and a great sense of emotional instability and turbulence. Whether the day is experienced as ‘good’ or ‘bad’, may depend entirely on subtle events, but the emotional consequence of those events may be impactful. Particularly for men, the anger and frustration toward others and drastic outbursts was something that could change in an instance (quote 1E).

However, as some of the participants describe, they also show the possibility of laughing, in addition to having a sense of humor and self-irony - even when considering their own suicide (quote 1G). Furthermore, there seems, in some cases, to be an enhanced feeling of sensitivity, as exemplified in quote 1F, where a woman describes her experience of taking a shower. Here a sense of bodily sensitivity made it unbearable for her to even touch the water.

### 4.2. The biological (embodied) dimension: embodiment and agency

Common for the participants is that they describe every action, even minor tasks, as insurmountable. As one participant described it: “Even something so banal as getting a glass of water. It is such a big task, that you would rather sit and stare into nothing.” (Woman, 44, quote 2C). Without exception all the participants experienced great difficulties in for example just getting out of bed in the morning (quote 2A).

The participants describe how they experience their own body as both ‘heavy’ and ‘rigid’, limiting their actions as the body is ‘petrified’. While the participants experience that they are unable to act and control their own body, they describe that they feel intense awareness of their shortcomings and inabilities. They feel a tension between bodily abilities and their intentions, hopes, aspirations, and desires. As one woman describes it: “I want life, that’s what the struggle is about, but on the other hand, I just do not have the strength for it.” (Woman, 33, quote 2B).

When asked about their thoughts and feelings during their morning routines, the participants described their actions as huge tasks (quote 2C). Tasks that one ‘should’ and ‘ought to’ do, but which they experience they could not do (quote 2D). The participants also described actions that they were still able to do such as getting up to make breakfast for their children and getting them to school (quote 2E).

As exemplified in 2F and 2H, most of the participants also describe how even in the toughest periods of the depression, they found the agency to write suicide notes in addition to actively spending hours researching and planning their suicide. Through these suicidal thoughts and actions, one participant describes a glimmer of hope “that [the depression] will pass, and it would not come back.” (Woman, 32).

### 4.3. The psychological dimension: self-experiences and narratives

Many of the participants describe how they have extremely high self-expectations, and what they themselves see as an extremely idealized self-image. As in quote 3A, the participant wants to be a ‘good’ person, better than others, therefore, even in his toughest periods, he was always able to leave his apartment, and show up at university. When asked how he was able to do so, he describes that it “is because, I think, that one has to. There are some expectations that I must meet. And these expectations aren’t even that bad.” (Man, 24, quote 3B). Throughout the interview the participant both describes that the “expectations that I have of myself, it is those that are distorted” and that the expectations “aren’t that bad to live in compliance with.” He also describes how he perceives himself as being better than others, while saying that his ideals of how to be a ‘good’ person was “completely unrealistic” and that he is “the worst person in the world, and it would be better, if I was not here.” (Man, 24).

In addition to their self-image the participants describe the idea of being perfect and doing everything perfectly. This is typically described in relation to failure and how the participants think ‘one should be’. Be it work or social interactions, if something goes amiss, their world literally crumbles, as described by the participant in quote 3C. In addition, the participants continuously describe how having a certain self-worth and value is tied to work (quote 3D), accomplishments, and performance (quote 3F). When the participants are then not able to perform, accomplish, or do specific things, they describe the feeling of being worth nothing, a ‘bad’ person, and they feel shame.

The participant in 3E describes a conflict between how he sees himself and what he expects of himself. Because of this self-conflict, he would then rather not go out into the world, since his self-ideals would be challenged. He isolated himself, which left him increasingly lonely, since social interactions was also “very indispensable” for him. As this example shows the participants’ self-image are described as interwoven with the social world. In a social context they typically describe themselves as ‘bad’, ‘rotten’, ‘unable to be empathetic and present’, and they feel disconnected. One participant describes how, “In my head, people have a lot of thoughts about how bad of a person I am. And everything that I do is wrong. It is a feeling of never being able to be myself, and quickly overthink and overanalyze even tiny things.” (Woman, 33, quote 3G). Though when asked about her parents-in-law, she responds, that “there I feel, much more, that I can be myself, that I, much more, can be the one that I want to be.” (Woman, 33).

### 4.4. The social dimension: social relations

The experience of, and relations with, other people are described by the participants as being highly important to them, both in a negative way, when they compare themselves to others and in a positive way as a source of joy and happiness.

For example, when isolated and by themselves, the participants constantly think about other people. Either under the covers of the duvet (quote 4A) or just outside the door (quote 4B). This influences how the participants think of themselves, since they all have a strong feeling of being obligated to be someone specific. In quote 4C, the participant feels that he has to live up to society’s expectations and be a certain way for others. The participants describe that these obligations take their toll on them, since they in most cases feel as though they cannot live up to these expectations and are not worth anything compared to others. Generally, they feel that they are a huge burden to other people.

Even in what are seen for many of the participants as the toughest and most isolated time, when attempting suicide, their social relations take up a lot of space in their thoughts. In quote 4D, the participant was on the brink of suicide, and he took the time to tell his dearest how it was not their fault at all. Another example, 4E, shows similar thoughts. In fantasizing about her own funeral and in planning her suicide, she is very aware of the social aspects, making sure that no one is traumatized by her suicide, making sure the right people get suicide notes, and that her body can be used for organ-donations, so her death is not a social burden.

So, on the one hand, the participants describe how they experience social relations as part of problem and the reason why they are struggling. For example, the participants all compare themselves to others, and in this comparison, they describe how others will look down upon them, since they are not able to perform in the same manner as other people. That is even seen in close relations, such as in the case of friendship, described in quote 4F. On the other hand, the participants also describe, without exception, how social relations are highly meaningful to them and might help them out of their depression (quote 4H). For example, the participant from quote 4G describes her three grandchildren as her “three happy pills, and to do something with them, can really distract my attention, and let me be in something, that is filled with happiness and joy.” (Woman, 57).

## 5. Discussion

In the discussion we’ll start by addressing the challenge of patho-description in relation to the results and show how dealing with this challenge discloses nuances to the current phenomenological model of depression. We will unfold these nuances and further discuss what this means for advancements within the clinical work on depression.

### 5.1. The challenge of patho-description in depression

If we start by looking at the descriptive style and narratives that the participants use when they describe their experiences, we see examples where depression influences how they access and describe their own experiences. This is possible to see with the help of the phenomenological interview, since its specific methodology helps the participants in some instances to describe their experiences in more detailed and nuanced ways. This means that while the participants do retell and reproduce the same general, negative stories about depression, which corroborate the current phenomenological model of depression, they also provide new descriptions that differentiate from - and seem to conflict with - some of these general, negative stories.

For example, the participants describe their experience as permanent, full of pervasive feelings of guilt, shame, hopelessness, nothingness, meaninglessness, and apathy, but they also describe that they experience a variety of different feelings such as anger, fun, and bodily pain. These descriptions are consistent with one of the criteria in the DSM-5, namely that depressed mood must be present for most of the day, in addition to being present nearly every day. However, in DSM-5 the nuance of the emotional variation is underplayed, and the narrative primarily focuses on the negative aspects of the depressive mood.

In another example, the participants describe that their body is felt as heavy, rigid, and petrified and that they are unable to do even the smallest tasks of, e.g., getting a glass of water. At the same time, they also describe examples (e.g., take care of their grandchildren or planning their suicide) where they experience a lot of agency, and are able to do many different things.

When it comes to their self-experience and self-image they describe in a pessimistic way, how they see themselves as ‘bad’, guilty people, with no self-worth. At the same time, in an overly optimistic way, they also describe having high self-expectations and self-worth. They see themselves as the ‘best person’ in the world and want to live their lives in a perfect idealized way.

Lastly, they describe difficulties in social relations and how they isolate themselves. At the same time, they describe in a positive manner how important and meaningful their social relations are to them, even in their darkest moments (e.g., planning and attempting suicide). These descriptions resemble the paradox described by David Karp. People with depression have a desire to connect with others, and such interpersonal relation could help them regain their sense of belonging to the world. It becomes paradoxical, because the interpersonal connection that they desire and which could help them, they are at the same time unable to realize (Karp, 1996).^4^

How is it possible for the participants to maintain such seemingly opposing descriptions? Applying the phenomenological distinction between ‘pre-reflective’ and ‘reflective’ self-awareness to analyze the interview data, we see that the participants shift between these two different experiential levels, when they describe their experience. The pre-reflective level refers to the experiential life that a person lives through without self-reflecting upon her experience, whereas the reflective level refers to self-awareness where she reflects on the experiences, that she lived through. The difference between the two levels in the context of depression is between how people with depression experience the world through depression (pre-reflective level) and how they reflect on their experience of depression (reflective level).

At the reflective level they retell and reproduce the same general, negative stories about themselves and their experiences. Here they describe their experiences using somewhat technical concepts such as ‘apathy’, ‘shame’, ‘narcissistic’, and ‘self-evaluation’. Concepts that health-professionals and researchers would use. They likewise use concepts that are commonly used to described depression such as ‘nothingness’, ‘meaninglessness’, ‘dark’, and ‘emptiness’.

At the pre-reflective level, however, we see the nuances to such descriptions as exemplified above. When referring to this level, people with depression describe their daily actions, use vocabulary applied in daily conversations, and describe a variety of feelings, a type of agency, positive self-image, and positive social relations.

The experiential differences at the two descriptive levels, become apparent in the interview, when participants shift from using ‘I-descriptions’ to the use of ‘one-descriptions’, e.g., “I think that one has to” (quote 3B), or “So I feel society does a lot for me, so one has to give everything back, that one can” (quote 4C). Here we see the tension between descriptions at the pre-reflective level, where the participants describe how ‘I’ experience daily life, and the reflective level where they describe their experiences in explanatory ways of how ‘one’ should experience. This is one example of patho-descriptions, where depression influences the participants’ ability to access and describe their own pre-reflective and lived experiences. They describe depression as how one ought to experience it, where they reflectively ‘clean up’ many of the experiential nuances and zoom in on certain specific experience, which they then overgeneralize to create a simplified narrative about their depression.

One of the underlying drivers of patho-description in depression comes from what Fuchs (2019) calls an ‘existential defense mechanism’. In relation to the *Typus Melancholicus*, Fuchs describes this mechanism as a way for people with depression to protect themselves from their own vulnerability by striving for continuous harmony, social concord, and delivery of duties. This means that, in the interviews the defense mechanism may urge the participants to play the role of the ‘depressed person’ and tell stories and create narratives in a way, that they believe is expected from them.

The integration of a phenomenologically informed framework makes us able to deal with the challenge of patho-description by adding depth and richness to the descriptions, and disclosing descriptive nuances. In the following, we will unfold the descriptive nuances found in the interview results.

### 5.2. Descriptive nuances

We will unfold the descriptive nuances seen in the interview by discussing it against the current phenomenological model of depression as presented above. If we start by looking at the existential dimension in the model, we see that on a pre-reflective level people with depression experience a variety of different negative (e.g., shame, guilt, anger, bodily pain) and positive (e.g., joyful and humorous) emotions. Such a variety of feelings need to be added to the description of depression seeing as only overly negative experiences are currently mentioned in the phenomenological model. People with depression experience a variety of feelings and these vary throughout the day, which can be seen as an emotional flux in their otherwise stagnated stupor. At the reflective level, when they describe their experience of depression, such emotional variation and flux are typically neglected, and the experience of ‘meaninglessness’ and ‘nothingness’ is described as permanent and pervasive.

Fuchs (2013) emphasizes that there are of course emotions (e.g., guilt, despair, and anxiety) that remain despite the loss of affectivity, and he characterizes these emotions as having three distinct features: (1) They separate the person from the world and other people, rather than connect them, (2) their bodily felt quality is that of corporealization, and (3) they are embedded within a prevailing depressed mood. As we will show below, the interview results do not support such interpretations of the remaining feelings in depression. It is not possible to make such a clear demarcation as seen in Figure 1 of the ‘normal’ and the ‘depressed’ experiences and feelings. In fact, it is the continuous retention of the ‘normal’ experiences and the normative social world that help drive the emotional disturbances in depression.

To unfold this claim, we will discuss the biological (embodied) dimension in the phenomenological model of depression. In the interview results we do see a body-schematic disruption, where the field of affordances is distorted, sensorimotor inhibition takes place, and people experience an impaired sense of agency. However, at the pre-reflective level the participants also describe specific actions that they are still able to do such as picking up grandchildren from nursery or planning their own suicide.

To understand how it is possible for people with depression to retain agency we should differentiate between what De Haan et al.’s (2013) calls a global ‘landscape’ and a local ‘field’ of affordances (see also Rietveld and Kiverstein, 2014). The landscape refers to all the possible affordances that are potentially open to a particular form of life, such as humans or animals. In contrast, the field refers to the affordances open to a particular individual with her skills and interest in a specific time and space. So, the landscape might afford dancing for all members of the human lifeform, but it might not be available to all individual members at all times and places (e.g., an infant, person living with physical disability, or an elderly person).

Applying the distinction between ‘landscape’ and ‘field’ of affordances, Krueger and Colombetti (2018) argue for a difference between affordances in schizophrenia and depression. There are experiential disturbances of the individual’s field of affordances in both schizophrenia and depression, but schizophrenia differs by involving a global disturbance of the ability to access the shared landscape of human affordances – to access reality as a whole. Krueger and Colombetti (2018) do not elaborate on possible disturbances in the global landscape of affordances in depression.

However, if we look at the interview results, we see that in contrast to schizophrenia, there does not seem to be a disturbance of the ability to access the global landscape in depression, but a disturbance of *how* to access. In contrast to most accounts of depression (Rowe, 1978), which mention the complete alienation or isolation from the world, we see that the participants describe how they perform many different daily actions such as cleaning, doing the dishes, getting out of bed, or helping their kids. However, most of these daily actions are experienced as ‘tasks’, ‘performances’, ‘something to overcome’, and something they ‘must’, ‘ought to’, and ‘should be able to’ perform. This gives the participants a feeling of alienation from the world, but it is not complete isolation, as the world is still calling for action.

This means the global landscape of affordances is still accessible to people with depression, and the field of affordances solicits daily actions. However, the field does so in a specific way. The local affordance of, e.g., getting a glass of water (quote 2C) is not just experienced as a basic action impossible to do, as described by other accounts of depression (e.g., Karp, 1996). Rather, the local affordance is globally imbued with overarching normative demands of how the person ‘must’, ‘should be able to’, and ‘ought to’ perform the action. This colors the experience of the actions with the value of being a ‘task’, ‘struggle’, or ‘performance’. To include such understanding of affordances into the phenomenological model of depression would require another illustration than seen in Figure 1.

### 5.3. Revising the model

**Figure 2:**
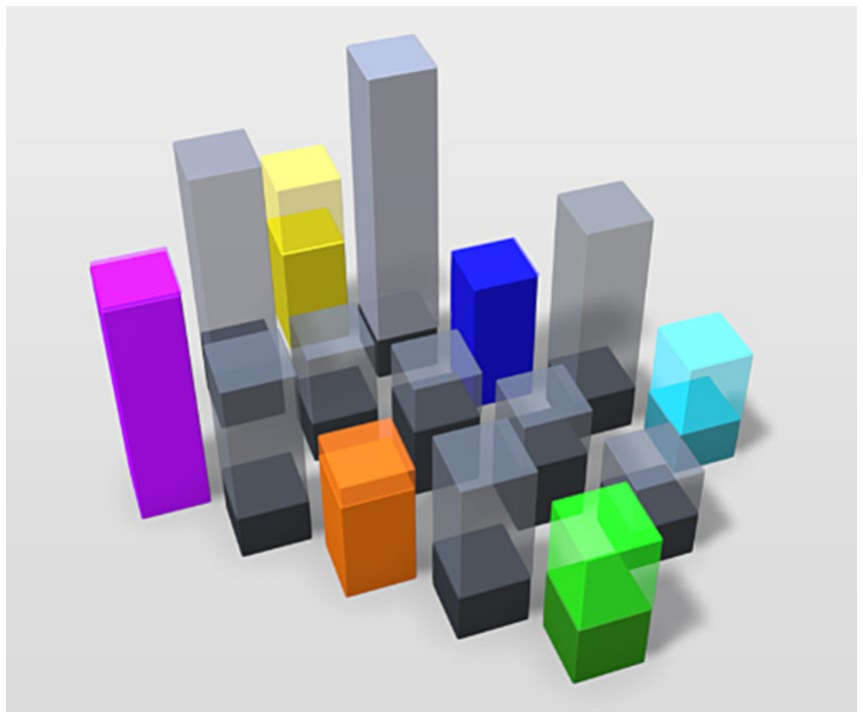
Illustration of the field of affordances in depression. The different colors and heights of each column illustrate how emotional variation and flux provide a differentiated field of affordances in depression with difference in relevance and salience. The difference in solid and transparent aspects illustrates how the global (transparent) affordances affect the local (solid) affordances.

In Figure 2 we have developed a nuanced illustration of the landscape and field of affordances in depression by combining the ‘normal’ and the ‘depressed’ experiences and feelings.

In adding different colors to the field, we empasize the emotional variation and flux that drives the affordances in depression. This includes the feeling of loss of feeling (grayness), and negative and positive feelings (color). The difference in height illustrates that affordances in depression retain a difference in relevance and salience. However, we use the difference in solid and transparent aspects of the boxes to emphasize the difference in the landscape and field of affordances. This shows how the global (transparent) affordances affect the local (solid) affordances, imbuing them with normative demands and expectations, diminishing the affective pull of the local affordances.

To further elaborate on this understanding of affordances in depression we will turn to the psychological dimension of the phenomenological model of depression. Here we see that the simple act of getting a glass of water becomes something that for people with depression reflects directly back on their identity and seeks to confirm the value of their self-worth. As many of the participants describe, they experience that their ability to perform their daily actions reflects how ‘good’ or ‘bad’ of a person they are. As such their daily actions are related to self-experiences of inability, shortcomings, blame and shame, when they are not able to do the actions. This means that almost every action becomes a stage where the individual must perform and show their self-worth, which is, undoubtedly, exhausting, and takes a huge toll on them.

At the reflective level we see that the participants describe their self-image in a pessimistic way, and because they cannot perform many of their daily actions, they see themselves as ‘bad’, guilty, with no self-worth. However, at the pre-reflective level many of the participants describe their self-experience in an overly optimistic way, with high self-expectations of their abilities and related self-worth. Their self-experience is colored by a performative stance of perfectionism. Due to this stance, the local affordances therefore represent ways in which to either prove or disprove one’s self-image, identity, values, and worth. With the high level of expectation and perfectionism, many of their actions will be failures, since their unreasonable rigid understanding of being a ‘good’ person allows only for unachievable self-expectations to be present. The consequence is that their self-narrative and self-image is created through their failures, which in effect makes them see themselves as ‘bad’, guilty, and with no self-worth.

Seeing as the ‘I’ in relation to itself is threatened in depression, one way to think about the experiences of corporealization is as another manifestation of the existential defense mechanism. Corporealization is an unintentional way for people with depression to protect themselves, since it makes it impossible for them to act. The system as a whole shuts down, disrupting their embodied and cognitive engagement with the world. In this way they cannot continue their attempts to act, which would only continue to disprove their own identity, value, and self-image.

If we focus on the social dimension of the phenomenological model of depression, we see that the participants show an extreme adherence to a performative society and an overidentification with such social norms and values. These precise, rigid ways to be, and the expectations to live up to in social and societal spheres, invoke strong feelings of obligation toward others and society. From their perspective, what is considered worthy and valuable is linked and always compared to other people. The performative task of how ‘one ought to’ act therefore becomes the only way to act, which is infused with social obligations. Unlike the general population, which is also influenced by social obligations and how other people see them, people with depression experience these social obligations to an existential degree. This means that it permeates their experiences in a way that disrupts their very ability to act and engage in the intersubjective and interaffective realm.

As claimed in the current phenomenological model of depression, it is typically believed of people with depression that they experience their social life as an alienating and isolated life, with no relationships. Contrary to this belief, we see at the pre-reflective level that the participants are in fact describing experiences of being hyperaware of, and hyper-sensitive to, their social relations. The hyper-social experiences are in many cases described by the participants as what fuels their feelings of immense guilt and shame. They ruminate extensively about social interactions they have experienced or future social interactions, since they desire to be perceived in a certain social light. Their experiences of sociality not only show how social life puts certain expectations unto people with depression, but also show the compassion they have toward others. In the case of planning suicide, people with depression cannot stand the thought of their nearest continuing to live without proper closure and reassurance that they were not at fault. They also plan their suicide, so that it will not be a traumatizing experience for anyone.

At a pre-reflective level, the interview results illustrate that the participants value their social life highly. At first glance, this seems to be contrary to Solomon’s (2014) description of his own social life in depression: “I did not care about love; about my work; about family; about friends…” (p. 59). However, at a reflective level, Solomon’s description still makes sense when compared to the participants’ experience. As the participant says in quote 3E: because he could not live up to his self-expectations in social interactions, he preferred not to engage socially at all. It’s the over-valueing and overwhelming pressure of social expectations in every interaction that makes people with depression physically isolate themselves and devalue their social life. This can be seen as another manifestation of the existential defense mechanism. They isolate themselves, not because they do not pre-reflectively value social interactions, but contrary, because they hyper-value social relations and sociality to the extent that it will only undermine and challenge their self-image and self-worth. In social isolation, they however continue to live a somewhat social life, ruminating on experienced or future social interactions.

## 6. Clinical advancements

We will end by looking at what it means for diagnostic and therapeutic advancements to incorporate these descriptive nuances into the phenomenological model of depression.

### 6.1. Diagnostic nuances

First of all, Ritunnano et al. (2023) have argued that there is an oversimplification of psychopathological descriptions incorporated in many of the manuals and rating scales used to classify, measure, and diagnose mental disorders. In the case of schizophrenia, Nordgaard et al. (2019) have for instance shown the challenge of working with self-rated questionnaires for ‘psychosis-like’ symptoms in the general population. They found that the use of self-rating scales resulted in 82.5% of the cases being false positives when re-tested against phenomenological interview.

In the case of depression, the symptom criteria seen in DSM-5, ICD-11, and the rating scales (e.g., Hamilton Depression Rating Scale, and Major Depression Inventory) incorporate the same oversimplified, negative, and general script of symptoms that the participants reproduce in their narratives, such as: “Have you lost interest in your daily activities? Have you had a bad conscience or feelings of guilt?” or asking if the patient have experienced a prevalent depressed mood (sadness, hopeless, helpless, worthless). What is important to emphasize here is that it is a categorical mistake to take the script and index of depression seen in the manuals and scales for the experience of depression itself (Kendler, 2016). This raises a diagnostic dilemma, namely whether the descriptions that people with depression provide of their symptoms are the experiences that they live through, or the descriptions they feel they ought to provide because they are listed in the manuals and scales and are the common stories about depression. If the latter is the case, it would potentially mean that due to the way we are currently diagnosing depression, we are overlooking experiential nuances that might be relevant for qualifying diagnosis. By putting too much emphasis on certain negative characteristics of depression, as listed in the manuals and scales, it could imply that we are currently over-diagnosing. Equally, since certain experiential nuances are missing from the manuals and scales, it likewise seems to call attention to the possibility of under-diagnosis.^5^

In regard to such diagnostic challenges in psychopathology, a number of scholars (Carel, 2012; Nordgaard et al., 2013; Fuchs and Koch, 2014; Nelson et al., 2018; Ritunnano et al., 2023) have emphasized the value of phenomenological psychopathology. The integration of a phenomenologically informed framework in psychopathology is able to add descriptive depth, richness, and nuance to clinical data, thus improving the identification of psychopathologies. It is our hope that by showcasing the methodological challenge and lack of nuances we found in our work on depression, we justify the claim that phenomenological psychopathology can help to improve diagnostic work. As such, it would be beneficial to add to the manuals and rating sales the experiential nuances we have found. For example, adding more nuanced questions about the variety of feelings and type of agency that exists in depression or about the experiences of positive self-image and hyper-social relations, which affects the self- and social experiences in depression in a negative way.

### 6.2. Phenomenological strategies

One consequence of our findings is that they problematize the uncritical use of traditional ‘talking therapies’ such as Cognitive Behavioral Therapy (CBT) for clinical treatment of depression. The issue is that such therapeutic treatment works at the reflective level of experience and might counterproductively strengthen the narrative that people with depression tell themselves and others. By continuously retelling the same general, negative story with a hyperfocus on negative characteristics, the patho-descriptions will potentially keep people with depression in a vicious and repetitive narrative circle.

One way to counteract this narrative effect in ‘talking therapies’ is to include both the descriptive nuances and the challenge of patho-description in the therapeutic work. One way to do so is by applying Carel’s (2012) phenomenological toolkit that helps patients to engage in reflective understandings of their own illnesses, disorders, and challenges. Such a toolkit helps suspend everyday routines and beliefs, and makes repetitive actions explicit, which could hopefully elicit, and potentially escape the challenge of patho-description. By incorporating a toolkit that apply phenomenological strategies to make patients aware of their own narrative challenges, CBT, and other “talking therapies” could both become a more effective therapy and a way to counteract the pathological effects that depression has on peoples’ self-narratives.

Another point we want to make, based on our findings, is that there are overlooked and untapped resources of agency in depression that can be used for therapeutic work. This is seen for instance in the case of suicide. Contrary to what a number of phenomenologically informed scholars (e.g., Benson et al., 2013; Fuchs, 2019; De Haan, 2020a,b) previously have claimed, in our findings the participants described at the pre-reflective level the idea, planning, and researching of suicide as a way for them to mobilize agency, energy, and social connection. For example, the same participant (quote 2F) that was unable to get a glass of water is able to spend a large amount of time writing suicidal notes, be active on online forums and the internet in order to research and plan her suicide.

One way to interpret this, is that the actions revolving around their suicide are not experienced as a ‘task’ and ‘performance’ they ‘ought to’ or ‘should be able to do’, and are not experienced with the same psycho- and sensorimotor inhibition as other daily actions. It is rather experienced as a way to break free from normative, societal, and self-expectations. As such, the act of writing suicide letters or planning their funeral becomes a way for them to socially ‘reconnect’ with others, where they do not risk the possibility of being rejected and disproven. Here they are able to re-synchronize, resonate, and feel the possibility of being reciprocated in their current situation, although it is with imagined others.

The example of suicide shows how there is a pre-reflective ‘opening’ in depression where people are capable of re-synchronizing and resonating with the world, and where it is possible to experience a horizon of possibilities, agency, and a reattachment through interaffectivity and intercorporeality. This ‘opening’ is also seen in specific social interactions, for example where one participant describes being together with her grandchildren as her ‘happy pills’. These examples show that it is possible to open up for specific types of action, agency, and social connection in depression. It needs, however, to be actions and social connections that do not fall into the normative and performative trap, from which people with depression are trying to escape.

To work at the pre-reflective level in clinical interventions and treatments is a phenomenological strategy used in Body-Oriented Psychotherapy (BPT). BPT is a clinical approach that rethinks clinical work on depression phenomenologically by working with the pre-reflective and embodied level of experiences (Röhricht, 2000, 2009; Koch et al., 2013; Fuchs and Koch, 2014; Galbusera et al., 2016). BPT uses nonverbal techniques to work with bodily resonances, body memory, and embodied affectivity. Types of psychotherapy like BPT, can be defined as what Maiese (2015) terms the ‘bottom-up treatment methods.” In contrast to more ‘top-down’, reflective psychotherapeutic and cognitive treatments, these methods work with the pre-reflective dynamics of bodily movements and affect, and they consist of therapeutic methods such as yoga, music, dance, and movement therapies.

In comparison to many versions of top-down methods (e.g., CBT), which are highly individualistic (e.g., one-to-one) approaches, bottom-up treatment methods also emphasize the social aspects of psychotherapy. This can be seen in rethinking the method of ‘group therapy’ (Röhricht et al., 2014), but also by adding a community and family aspect to treatment, including the daily environment, partners, friends, and family, with which and with whom people with mental disorders are always interacting (De Haan et al., 2014). As we see in the interviews, the social interactions that people with depression engage in daily can in a non-performative way become the ‘happy pills’ that they need. It is crucial, however, that including social aspects of group therapy, community, or family into the treatment of depression, is done in a way that does not reproduce the performative and normative conditions that drives the experience of depression.

Based on the nuanced understanding of depression described above, it seems that phenomenologically inspired clinical approaches would be able to bypass the patho-descriptive challenge. In doing so such approaches experientially ‘open up’ at a pre-reflective level for agency and social connection. There is already evidence to support the use of phenomenological, bottom-up, and embodied strategies in clinical work on depression. For example, dance/movement therapy (DMT) has shown positive effects on people with depression by awakening their experience of joy and vitality, and by decreasing their negative feelings (Ritter and Low, 1996; Kipper and Ritchie, 2003; Koch et al., 2007). Also, music-therapy have been shown to lead to reductions–at least short-term–of both general (e.g., depression) and negative symptoms in schizophrenia (Talwar et al., 2006). Art therapies have also been used as complementary approaches to treatment of depression (Lehofer and Stuppäck, 2005), showing positive effects when people with depression engage with nonverbal modes of expression such as painting and sculpting, playing music, or theater.

## 7. Conclusion

In this article we have argued that the current phenomenological model overlooks experiential nuances highly relevant for work on depression. This is a result of the challenge of patho-description observable in depression. Based on our work with the phenomenological interview we unfold these experiential nuances and incorporate them in a revised phenomenological model of depression (e.g., in Figure 2).

In depression we have illustrated that, in order to protect the ‘I’ in relation to itself, different manifestations of an existential defense mechanism will take effect. For example, people with depression clean up their self-narrative and describe their experiences in a general, simplified, and negative way that overshadows their diverse and varied emotional life. A sense of agency is still present in depression, but experiences of corporealization will kick in, making it impossible for them to act and disprove their own identity, value, and image. They will also isolate themselves socially, not because they do not value social interactions, but contrary, because they hyper-value social relations and sociality to the extent that it undermines their self-image and self-worth.

Highlighting such experiential nuances, we believe not only strengthens the phenomenological model of depression, but it is also a testimony to the clinical advancements in work on depression that phenomenological psychopathology provides. Firstly, the current manuals and rating scales used to diagnose depression incorporate the same oversimplified, negative, and general script of symptoms that the participants reproduce in their narratives. Due to the challenge of patho-description, the manuals and scales overlook experiential nuances that are necessary for qualifying diagnostic work in depression. As we have shown, a revised phenomenological model of depression will be able to add the needed depth and nuance to diagnosis of depression.

Secondly, as we have argued, the uncritical use of traditional ‘reflective and talking therapies’ for clinical treatment of depression may counterproductively boost the self-narrative in depression. The different manifestations of the existential defense mechanism in depression can in fact complicate the clinical treatment of depression. As we have shown, phenomenological, ‘bottom-up’, and ‘body-oriented’ approaches to psychotherapy is one way to bypass this mechanism. At a pre-reflective level, these approaches will ‘open up’ for a sense of agency and social re-connection.

## Data availability statement

The original contributions presented in the study are included in the article/supplementary material, further inquiries can be directed to the corresponding author.

## Ethics statement

Ethical approval was not required for the studies involving humans because according to the Helsinki Declaration of 1975 (revised in 2008) and Danish ethics committee law §2, no. 1, ethical approval is not required for questionnaires, qualitative interviews and non-health scientific intervention studies, which concerns our study. The studies were conducted in accordance with the local legislation and institutional requirements. The participants provided their written informed consent to participate in this study. Written informed consent was obtained from the individual(s) for the publication of any potentially identifiable images or data included in this article.

## Author contributions

All authors listed have made a substantial, direct, and intellectual contribution to the work and approved it for publication.

## Footnotes

^1^Thanks to one of the reviewers for making us emphasize this heritage.

^2^Thanks to one of the reviewers for emphasizing this point.

^3^We are critical about the way in which Figure 1 differentiates the ‘normal’ and the ‘depressed’. Firstly, what is meant by ‘normal’? Secondly, we do not believe, as we will argue below, that it is possible to make such clear demarcation between the ‘normal’ and the ‘depressed’. Instead, we will suggest another illustration of the field of affordances in depression, as seen in Figure 2.

^4^Thanks to one of the reviewers for making us aware of this paradox and reference.

^5^Thanks to one of the reviewers for making us emphasize the potential problems of over- and under-diagnosis in this context.
